# Editorial: Understanding the effects of metabolites and trace minerals on microbes during infection

**DOI:** 10.3389/fcimb.2023.1276271

**Published:** 2023-08-30

**Authors:** Thomas Naderer, Andre Mu, Andrew J. Monteith, Tania Wong Fok Lung

**Affiliations:** ^1^ Biomedicine Discovery Institute, Department of Biochemistry and Molecular Biology, Monash University, Clayton, VIC, Australia; ^2^ Host-Microbiota Interactions Laboratory, Wellcome Sanger Institute, Hinxton, United Kingdom; ^3^ European Molecular Biology Laboratory (EMBL)– European Bioinformatics Institute, Hinxton, United Kingdom; ^4^ Department of Microbiology, University of Tennessee, Knoxville, TN, United States; ^5^ Department of Pediatrics, Columbia University, New York, NY, United States

**Keywords:** immunometabolism, host-pathogen interaction, bacterial metabolism, microbial metabolic activity, microbiota-derived metabolites, itaconate, adenosine, metabolic cross talk

## Introduction

Successful microbial pathogens establish infection and subvert the host immune response by utilizing their virulence determinants. Perhaps more importantly, the survival and replication of these pathogens within the microenvironments of infection sites depend on their ability to utilize alternative nutrient sources. This often involves the rewiring of metabolic pathways. Changes in microbial metabolic activities were historically used to identify microbial species by gold standard biochemical tests. However, over the last two decades, microbial metabolism has been neglected, particularly in favor of next generation sequencing.

The emerging field of immunometabolism, the intersection between host cellular metabolism and immune function, has reignited interest in microbial metabolism. This is because the metabolic reprogramming of host cells not only alters immune responses but also the nutritional environment that supports microbial growth. Conversely, microbial metabolism affects immune cell function by depleting immunoregulatory metabolites. Outcomes of host-pathogen interactions are further affected by the diet and microbiota-derived metabolites, not only in the gut but also in distal organs. This Frontiers Research Topic highlights the metabolic cross-talk between the host and pathogen during infection, the factors that affect these dynamics, and their consequences on the infection outcome.

## Immunometabolism

**Figure 1:**
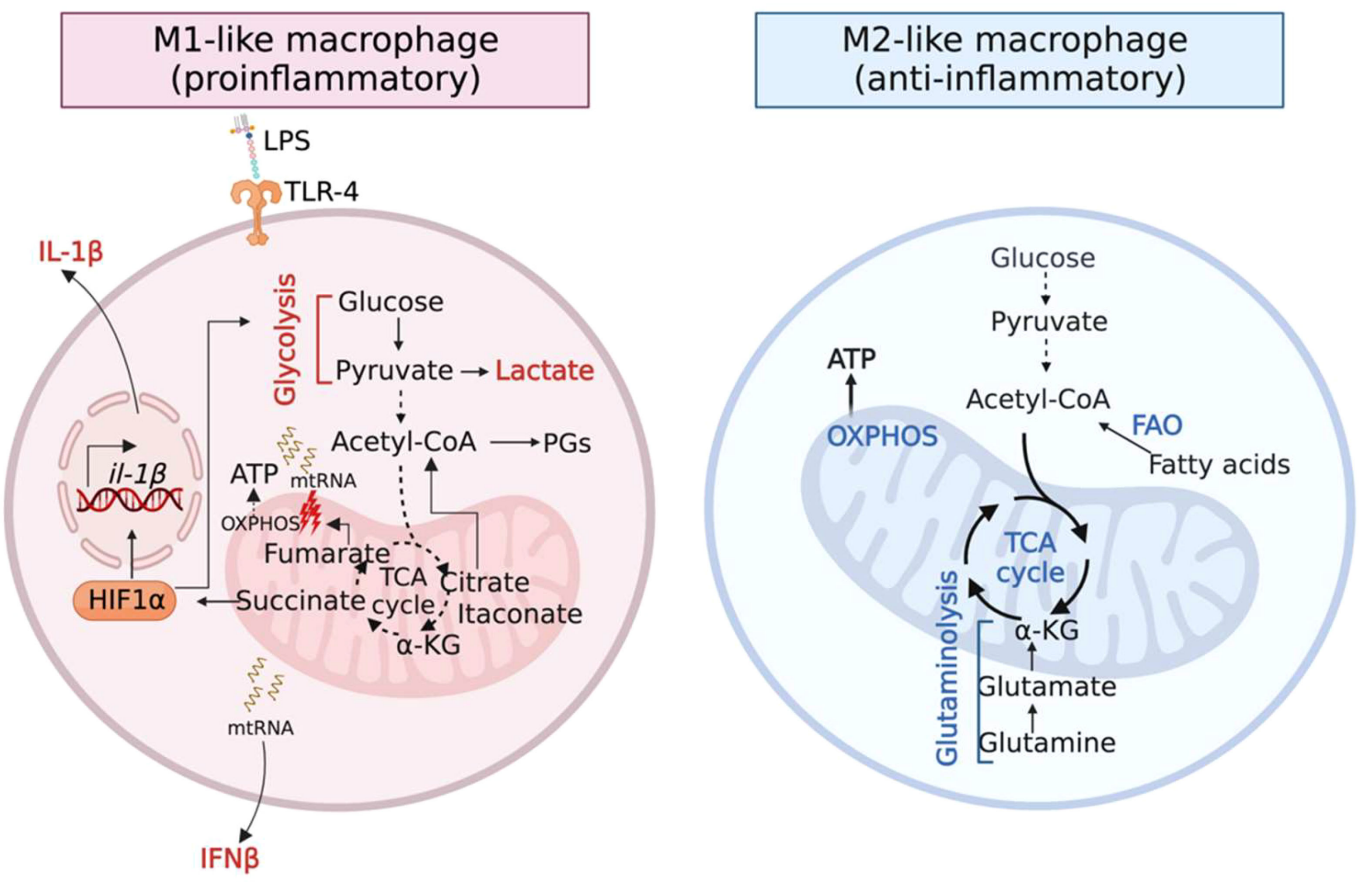
Metabolic activities govern the function of macrophages. (Left panel) Inflammatory (M1) macrophages accumulate the TCA metabolites citrate, succinate, fumarate and itaconate as a result of several breaks in the TCA cycle. Citrate, succinate and fumarate promote inflammation. Itaconate restores homeostasis via its anti-inflammatory and anti-oxidative properties. (Right panel) Anti-inflammatory (M2) macrophages downregulate glycolysis and upregulate metabolic pathways that fuel the TCA cycle, OXPHOS and the production of reactive oxygen species (ROS).

Immunometabolism is an emerging concept that is central to both innate and adaptive immune regulation. The activation of specific metabolic pathways not only generates energy (ATP) but also dictates the function of immune cells (O'Neill et al., 2016). One prime example is the metabolic reprogramming which macrophages undergo following stimulation with the bacterial immunogen lipopolysaccharide (LPS) that fuels their pro-inflammatory signaling and microbicidal properties (O'Neill et al., 2016) (**Figure 1** left panel). This involves switching from their basal metabolic state, mitochondrial oxidative phosphorylation (OXPHOS), to glycolysis. Several interruptions in the tricarboxylic acid (TCA) cycle are observed in LPS-stimulated macrophages, which result in the accumulation of the mitochondrial metabolites citrate, succinate, fumarate and itaconate and their export to the cytosol.

Citrate is converted to acetyl-coA that is directed towards the production of inflammatory lipid-mediators such as prostaglandins (PGs) (Infantino et al., 2011). Succinate stabilizes the transcription factor hypoxia-induced factor 1 alpha (HIF-1α), promoting glycolysis and the production of the pro-inflammatory cytokine IL-1β (Tannahill et al., 2013). Recently, fumarate was highlighted as a pro-inflammatory metabolite given its role in type I interferon (IFN) activation (Hooftman et al., 2023). Fumarate causes mitochondrial stress and damage, impairing respiration and releasing mitochondrial RNA (mtRNA), which induces the production of the cytokine IFNβ. Thus, the above-mentioned metabolites polarize macrophages to a pro-inflammatory M1-like phenotype. Following inflammation, itaconate is produced in the mitochondrial matrix by the enzyme aconitate decarboxylase 1, Acod1 (also called Irg1). Itaconate exerts anti-inflammatory and anti-oxidative properties to restore homeostatic balance (Peace and O'Neill, 2022).

In contrast, anti-inflammatory (M2-like) macrophages downregulate glycolysis and upregulate OXPHOS through catabolic pathways such as fatty acid oxidation (FAO) and glutaminolysis (Jha et al., 2015; Liu et al., 2017) (**Figure 1** right panel), which can also play a critical role in resolving infection. For example, recruitment of M2-like macrophages facilitates the transition into a post-inflammatory resolution phase that is critical to resolving staphylococcal skin/soft tissue infections (Thurlow et al., 2018). Interestingly, it is increasingly being recognized that microbial pathogens can subvert immunometabolism to promote infection (Yamada et al., 2020; Wong Fok Lung et al., 2022; Tomlinson et al., 2023).

## Impact of microbial metabolism on immunometabolism

Live microbial pathogens impose a profound metabolic stress on the host that can override the macrophage reprogramming associated with LPS or other pathogen-associated molecular patterns (PAMPs). For example, during pulmonary infection, multidrug-resistant *Klebsiella pneumoniae* induces an airway metabolic response distinct from that stimulated by the heat-killed pathogen or its purified LPS (Wong Fok Lung et al., 2022). This response is characterized by an increase in metabolic pathways that fuel OXPHOS, promoting immunosuppression and tolerance to infection, rather than glycolysis that is associated with the highly pro-inflammatory signaling necessary for bacterial clearance. In addition, the host metabolite itaconate is particularly prominent during infection with metabolically-active *K. pneumoniae*. Absence of this immunomodulatory metabolite abrogates host tolerance to *K. pneumoniae* infection and is instead accompanied by excessive inflammation and immunopathology (Wong Fok Lung et al., 2022). The role of itaconate and other anti-inflammatory metabolites such as adenosine in promoting immunosuppression in myeloid cells is discussed by Urso and Prince.

The Gram-positive bacterial pathogen, *Staphylococcus aureus*, employs a similar metabolic strategy to cause persistent infection. The ability of staphylococcal biofilms to dampen inflammation via the induction of immunosuppressive myeloid cells and their production of the anti-inflammatory cytokine IL-10 is attributed to *S. aureus* production of lactate (Heim et al., 2020). This metabolite inhibits histone deacetylase 11 (HDAC11) to enhance *Il-10* transcription. These highlight the crucial contribution of active bacterial metabolism in contrast to PAMPS in determining the outcome of the infection through metabolic cross-talks.

## Adaptation of microbial pathogens to metabolites

Whilst pathogens can manipulate immunometabolism to evade immune clearance, they must also adapt to the metabolic milieu to ensure their survival. Several metabolites and metabolic by-products that are produced by the host and microbial cells during infection are toxic, thereby imposing a strong selective pressure that drives microbial adaptation. Urso and Prince provide a detailed overview of how bacterial pathogens alter their own metabolism and transcriptional profile to optimize their fitness, particularly in response to the electrophile itaconate and to reactive oxygen species.

In addition, Mitchell and Ellermann focus on long chain fatty acids (LCFAs), derived from host and microbial cells as well as dietary sources, that act as signaling molecules and regulate virulence in enteric pathogens.

## Impact of microbiota-derived metabolites

Host-pathogen dynamics are further complexed by commensal microbes and their metabolism. For example, short-chain fatty acids (SCFAs), the end products of the fermentation of dietary fibers by the anaerobic intestinal microbiome, have been associated with positive outcomes following microbial infections as well as other inflammatory diseases. In a mini review, Zhang et al. summarize the beneficial effects of SCFAs on the immune response to sepsis-associated encephalopathy (SAE). These are mediated by their anti-inflammatory and antioxidant properties. Lopez et al. discuss the immunomodulatory and therapeutic effects of SCFAs in inflammatory bowel disease (IBD) and close the cycle of host-pathogen-microbiota interactions by examining host factors that alter the gut microbiota and their production of microbiome-derived metabolites.

## Conclusions

The articles in this Research Topic illustrate how the metabolic cross-talk between host, pathogen and microbiome affects the outcome of infection. The metabolic potential of microbes as a virulence strategy is being revisited in light of the more recent studies documenting the impact of microbial metabolic activity in shaping the immune response to *in vivo* infection (Heim et al., 2020; Tomlinson et al., 2021; Wong Fok Lung et al., 2022; Tomlinson et al., 2023). In addition, the ability of the bacteria to co-opt metabolites within the microenvironment enables their survival and adaptation.

## Author contributions

TWFL: Writing – original draft, Writing – review & editing. TN, AMu, and Amo: Writing – review & editing
